# Factors That Influence the Use of Dietary Supplements among the Students of Wroclaw Medical University in Poland during the COVID-19 Pandemic

**DOI:** 10.3390/ijerph19127485

**Published:** 2022-06-18

**Authors:** Anna Merwid-Ląd, Marta Szandruk-Bender, Agnieszka Matuszewska, Małgorzata Trocha, Beata Nowak, Marie Oster, Adam Szeląg

**Affiliations:** 1 Department of Pharmacology, Wroclaw Medical University, J. Mikulicza-Radeckiego 2, 50-345 Wroclaw, Poland; agnieszka.matuszewska@umw.edu.pl (A.M.); malgorzata.trocha@umw.edu.pl (M.T.); beata.nowak@umw.edu.pl (B.N.); adam.szelag@umw.edu.pl (A.S.); 2 English Division of the Faculty of Medicine, Wroclaw Medical University, T. Chałubińskiego 6a, 50-368 Wroclaw, Poland; marie.oster@student.umw.edu.pl

**Keywords:** dietary supplements, OTC drugs, stress, anxiety, depression, sleeping problems, Medical University students, COVID-19 pandemic

## Abstract

Background and aim: The use of dietary supplements (DS) and over-the-counter (OTC) drugs is increasing every year. The COVID-19 pandemic might additionally influence the use of such preparations. The study aimed to investigate factors influencing the use of dietary supplements (DS), including stress-relieving supplements, by the students. Methods: In the cross-sectional study, 624 students of the Wroclaw Medical University in Poland, from the second to the last year of studies, completed the anonymous questionnaire, consisting of 22 items, about the use of DS/OTC drugs during the academic year 2020/2021. Obtained data were analyzed using Pearson’s chi-square test, the U-Mann Whitney test, the Kruskal–Wallis test with the post-hoc analysis, and with logistic regression. Results: About 70% of students declared the use of any DS, 33% used DS for stress, anxiety, depression, or sleeping problems, and 59% used other DS. The most important factors influencing the decision to take any kind of DS were Division (*p* = 0.0001, odds ratio [OR]: 0.35, and confidence interval [CI]: 0.21–0.59), a self-estimated level of stress (*p* = 0.014, OR: 1.13, CI: 1.03–1.25), and self-estimated level of knowledge about DS (*p* = 0.0000, OR: 1.31, CI: 1.19–1.36). In the case of students taking DS for stress, anxiety, depression, or sleeping problems, the level of stress and the declared knowledge had the greatest impact on the decision for such a use of DS (*p* = 0.0001, OD: 1.24, CI: 1.11–1.39 and *p* = 0.0000, OD: 1.35, CI: 1.22–1.5, respectively). The COVID-19 pandemic did not change the pattern of DS/OTC drug usage in about 33% of students. Those who started taking DS during the pandemic accounted for 19% of all students. Conclusions: The use of DS is common among Wroclaw Medical University students with some differences between subgroups of respondents. Additionally, despite declared good knowledge about DS, most students declare the need to learn more about them.

## 1. Introduction

The World Health Organization (WHO) defines the quality of life (QoL) as the perception of a person of her/his position in life. Many factors are of importance in this regard, such as culture, value system, and relation to their standards, goals, and expectations. Physical, intellectual, emotional, or social factors may easily affect an individual’s QoL. Some important examples are diet, exercise, safety, activities, privacy, autonomy, social contact, and support [1,2].

Diet is a broad term and may be understood not only as the kind of food habitually eaten, but also as the intake of a variety of dietary supplements, not always correctly used. Over recent decades, dietary supplements (DS) and nutraceuticals have become widely available in most countries. The common belief that the natural origin of these products makes them safe and that they may prevent or treat diseases without adverse effects caused their sales to increase [3,4]. It is estimated that about 50–75% of the adult population uses a variety of DS sporadically or routinely [5,6,7]. After the outbreak of coronavirus disease 2019 (COVID-19), the use of dietary supplements increased, especially in the first period of the pandemic [6,8]. However, many people also used a variety of DS or OTC drugs during the second wave [9]. According to some databases available in Poland, the most common reason to take dietary supplements was to improve immunity, the second most common reason was for overall health and wellness, followed by vitamin D supplementation. It is also important that about one-fourth of respondents were taking the DS for stress management and more than 10% to improve sleep [6]. In other countries, the main reasons to take different DS were similar, for example, to be healthy, to meet nutritional needs or correct deficiencies, to improve immunity, to reduce stress, to relieve fatigue, or to improve concentration [7,10,11]. During the pandemic, there was a significant increase in the use of vitamin C, vitamin D [9,12], and zinc-containing products. These supplements are considered helpful in the defense against viral infections [12]. Irrespective of the COVID-19 pandemic, factors that influence DS intake vary greatly. Of great importance is the belief that taking dietary supplements prevents possible illness. The impact of modern media cannot be overestimated [13]. Of great note are some sociodemographic factors (e.g., age, gender, diet, lifestyle, income, or educational level) [14,15]. Additionally, some reports suggest an increase in awareness about health and increased intake of not only nutritional supplements, but also immunity-boosting food [16]. 

COVID-19 affected the everyday life of people all over the world. In most countries, self-isolation at home, online work and learning, social distancing, and closure of educational institutions including universities were ordered. Lockdown made access to the doctors much more difficult [17] which might have influenced the tendency toward self-medication. However, it is not a new phenomenon [18] and students often choose OTC drugs, because it saves their time and, very often, the cost of a visit a to a physician [19]. 

COVID-19 changed the dietary patterns and generated high levels of stress [20,21,22,23]. Students at medical universities cope with very difficult courses in a variety of majors of studies. Distance learning in Poland from March 2020 to December 2020 caused disturbances in practical training which was a very unusual situation and may have been a reason for unfavorable changes in lifestyle. Therefore, it is very important to identify factors that may have influenced these changes.

The presented study was designed to evaluate the use of DS among students of the Wroclaw Medical University during the academic year 2020/2021 (from October 2020 to September 2021) and how the pandemic changed the pattern of DS intake. Most studies in Poland and other countries concerning this problem, focused on the habits and their changes during the first outbreak of the COVID-19 pandemic. We would like to assess the use of DS, OTC drugs, or prescription-only medicines (POM) in the middle of the pandemic period. Attention was also focused on the self-assessed stress level and the use of DS for stress, anxiety, depression, or sleeping problems, as well as on the self-assessed level of knowledge about DS and opinions about the necessity to have classes about DS during the study. The obtained results should help in taking specific actions at the university, e.g., preventive or educational.

## 2. Materials and Methods

### 2.1. Design of the Study

A voluntary and anonymous online survey was conducted between 14 November 2021 and 23 December 2021. The groups of the respondents were the students of the Polish and English Division of Medical and Dentistry Faculties and the Polish Division of Pharmacy and Health Science Faculties (pharmacy, medical analytics, dietetics, physiotherapy, nursing, midwifery, and paramedics) at Wroclaw Medical University in Poland. To collect answers from the respondents, Polish electronic questionnaire and its English-language equivalents were prepared using Google^®^ Forms. The links to the questionnaires (Polish and English versions) were sent via the official university emails to the year representatives of all majors of the studies to be shared with students. 

The questions were about the academic year 2020/2021, therefore, the students were asked about their dietary habits and the use of dietary supplements during their first to the fourth or fifth year of the studies (depending on the major), respectively. The students from the second to the final year of their studies (fifth or sixth, depending on the major) were included in the study and invited to complete the survey. First-year students were excluded from the study, because during the academic year 2020/2021 they were in the final year of their high school, and they were learning in different conditions to university students. Moreover, some of the final-year high-school students in Poland are not yet of legal age, which excludes independent decisions regarding treatment options, taking medicines, or dietary supplements.

Due to the pandemic and sanitary regime at the Wroclaw Medical University, the online form was chosen, and the written informed consent was waived. In the invitation, students were informed about the voluntary character of the participation and the possibility to stop the questionnaire at any time. The questionnaire was designed and prepared by the authors of the study without the use of ready-made questionnaires, e.g., available online or presented in other papers. The survey was pre-checked first by two academic tutors (not involved in the creation of the survey) and later pre-tested on a group of 15 students (their answers were later not included in the analysis) to minimize the risk of ambiguity of the questions. Thereafter, the survey was kindly checked by the market research company worker. After making all corrections listed after the pre-check process the survey was approved by the Bioethics Committee and sent to the students.

### 2.2. Ethical Approval

The study was approved by the Bioethics Committee of the Wroclaw Medical University (agreements KB-765/2021 and KB-1017/2021) and conducted according to the guidelines of the Declaration of Helsinki.

### 2.3. Organization of the Questionnaire

The questionnaire contained 22 questions in 6 sections. In Section 1, consisting of 6 questions, the students were asked about gender, major, and year of studies. The respondents rated their perceived stress level and quality of diet during the academic year 2020/2021, and they answered the questions about the use of DS or over-the-counter (OTC) drugs for stress, anxiety, depression, or sleeping problems. Section 2 was addressed only to persons who did not take any DS or OTC medications for stress, anxiety, depression, or sleeping problems, and they were asked to provide their reason for not taking these products. Section 3 contained 8 questions about the detailed use of DS for stress, anxiety, depression, or sleeping problems (e.g., type, frequency, changes during the pandemic, place of purchase, method of recommendation, assessment of efficacy, and occurrence of adverse effects). Two questions in Section 4 were about the use of POM for stress, anxiety, depression, or sleeping problems and the use of DS or OTC medications other than for stress, anxiety, depression, or sleeping problems. Section 5 was dedicated to students who took other DS or OTC drugs and contained questions about the reason for use and the type of dietary supplements. The last section, Section 6, consisted of 3 questions, and respondents were asked to rate their knowledge about dietary supplements and if they had learned about them during their studies. The last question was addressed to students in the final year and concerned the need to include the topics related to DS during the program of study. The detailed English-language version of the questionnaire constitutes Attachment S1 and may be found in Supplementary Materials.

### 2.4. Statistical Analysis

Statistical analyses were performed using TIBCO STATISTICA 13.3 PL (StatSoft, Kraków, Poland). Pearson’s chi-square test was used to compare the differences in categorical variables between the subgroups of respondents. After excluding the normality with the Shapiro–Wilk test, the U-Mann Whitney (for two compared groups) or Kruskal–Wallis tests with Dunn’s post-hoc test and Bonferroni correction (for more than two compared groups) were used to analyze differences between groups in the year of the studies, perceived level of stress, and the level of knowledge about DS (expressed in linear scale) in subgroups of students. Logistic regression was performed to assess the impact of analyzed features (independent factors) on the of use any DS, DS for stress, anxiety, depression, or sleeping problems, DS other than for those indications, and the use of POM (dependent factors). A *p*-value < 0.05 was considered statistically significant.

## 3. Results

### 3.1. General Characteristics of the Population

At Wroclaw Medical University in Wrocław, Poland, medicine, dentistry, pharmacy, medical analytics, and physiotherapy are uniform master’s studies, whereas dietetics, nursing, midwifery, and public health are 3-year bachelor’s and 2-year master’s studies. Paramedics is only a 3-year bachelor’s program. Medicine is a six-year program (12 semesters) whereas pharmacy is an 11-semester program of study. To simplify the discussion and statistical analysis, students of the 1st year of second-degree MA studies were considered as 4th years, and students of the 2nd year as 5th year students. More detailed characteristics of the students based on the year of the studies is presented in Supplementary Table S1. 

**Figure 1:**
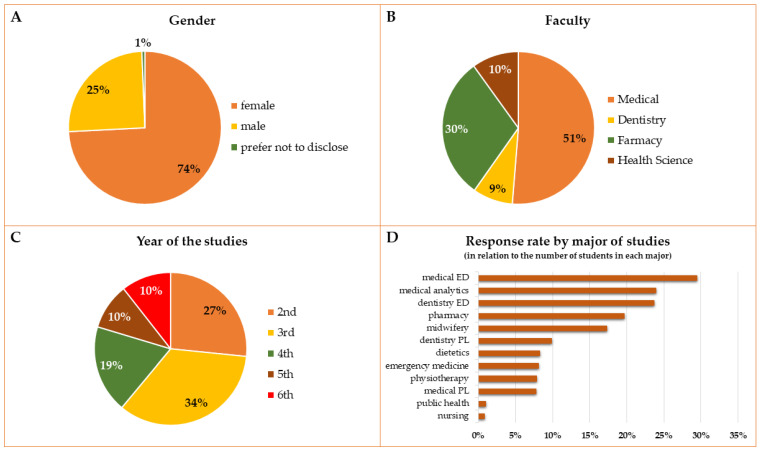
General description of the respondents. Gender (**A**), Faculty—Medical and Dentistry Faculties conducted studies in Polish and in English language (**B**), year of the studies—the survey was about the academic year 2020/2021, therefore, it did not include students from the 1st year (**C**), response rate by majors of the studies concerning the number of students in each major (**D**).

The survey was about the academic year 2020/2021, therefore, students from the 1st year were excluded and only students from the second to the final year were the respondents to our questionnaire because the period to which the questions pertained corresponded to their first to fifth year of the study. According to the data from the Dean Offices (as at 23 October 2021), there were 4914 students from the second to final year at Wroclaw Medical University, and 624 of them took part in the survey and answered the anonymous questionnaire on the Internet. The overall response rate was 12.7%. Most of the respondents were women and the difference was significant considering Division (*p* = 0.0004) and Faculties (*p* = 0.0000). In the Health Science Faculty, only 9.7% of respondents were men, corresponding to 3.8% of all male respondents, whereas men from the Medical Faculty accounted for 67.5% of male respondents. Over 50% of students were from the Medical Faculty and the highest response rates were noted among the Medical and Dentistry English Division majors. Students most willing to answer the survey questions were the students from the 3rd, followed by those in the 2nd and 4th year of their studies. The general characteristics of the population are presented in Figure 1A–D. 

### 3.2. The Declared Type of Diet and the Overall Use of Any Dietary Supplements or OTC Drugs

**Figure 2:**
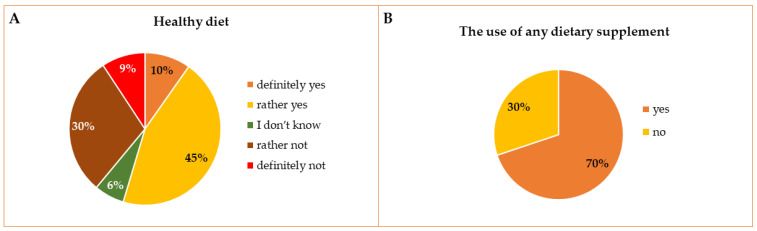
Declared type of diet (**A**), the overall use of any dietary supplements in the group of respondents (**B**).

Respondents assessed if their diet during the last academic year 2020/20201 was healthy. Most students referred to their diet as rather healthy, but 30% of students responded that their diet was rather unhealthy. A similar number of students referred to their diet as definitely healthy (61 persons) and as definitely unhealthy (58 persons). Declaring a healthy or unhealthy diet did not depend on the Division or Faculty, but a healthy diet was more often declared by students at a higher stage of their studies (64.6% vs. 52.1% for clinical and preclinical levels, respectively, *p* = 0.0119). The declared type of diet and the overall use of any dietary supplements are presented in Figure 2A,B.

Among all respondents, the use of any DS was declared by 70% of students. More willing to take dietary supplements were students of the Polish Division, women, Pharmacy students, students in later years, and students who considered their diet as healthy assessed their level of stress and level of knowledge about dietary supplements as high. Detailed comparisons between groups of students are presented in Table 1.

### 3.3. The Self-Assessed Level of Stress, the Use of Dietary Supplements, OTC Drugs, and Prescription-Only Medicines (POM) for Stress, Anxiety, Depression, or Sleeping Problems

**Figure 3:**
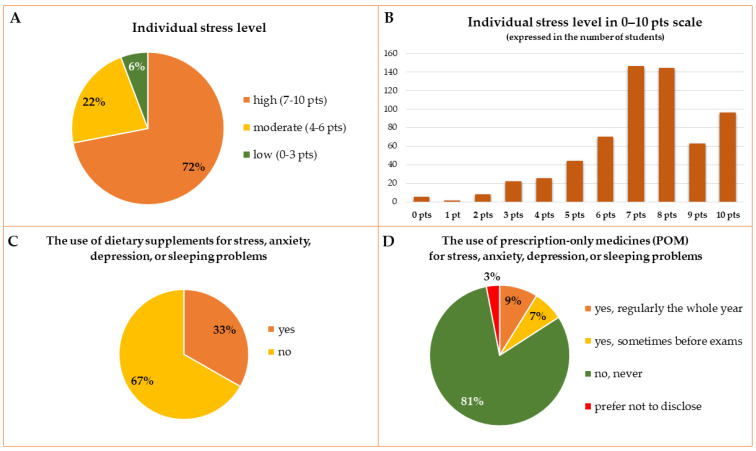
Individually declared general type of a diet during the academic year 2020/2021 (**A**), perceived level of stress (0—no stress at all, 10—extremely high level of stress) expressed in the number of students (**B**), the general use of dietary supplements or OTC drugs (**C**), and prescription-only medicines (POM) (**D**) for stress, anxiety, depression, or sleeping problems.

The perceived stress level was assessed by the students on a 0–10-point scale (0—no stress, 10—extremely high level of stress). Most students rated their stress level at 7 or 8 points (146 and 144 persons which account for 23.4% and 23%, respectively). For the statistical analysis, 0–3 points were considered as low, 4–6 points were considered moderate, and 7–10 points were considered a high level of stress. Most students assessed their stress during the last academic year as high (67.5%, 75.5%, 77.8%, and 74% of students from the Medical, Dentistry, Pharmacy, and Health Science Faculties, respectively). The perceived stress level is presented in Figure 3A,B. Irrespective of the Division, students declared similar stress levels and there were no significant differences in the percentage of students assessing their level of stress as high. However, the mean level of stress was significantly higher in the group of respondents from the Pharmacy Faculty when compared to the Medical Faculty students (*p* = 0.006), and significantly lower in the group of students from the 6th year when compared to the 2nd year students (*p* = 0.042). In the group of students assessing their diet as healthy, their mean level of stress was lower than in the group of students whose diet was unhealthy or difficult to assess (*p* = 0.0000). The detailed mean stress level in different subgroups of respondents is presented in Supplementary Table S1. 

Among respondents, 207 persons (33%) declared the use of DS or OTC drugs for stress, anxiety, depression, or sleeping problems during the academic year 2020/2021 (Figure 3C). There were 164 women and 43 men taking these types of products. The decision about taking the DS for stress, anxiety, depression, or sleeping problems did not depend on the Division, Faculty, gender, year of the studies, or declared type of diet, but depended on the self-assessed stress level and the self-assessed knowledge about DS. Students with high levels of stress took these kinds of DS or OTC drugs over two times more often than students with a low level of stress. Students who declared a low or moderate level of knowledge about DS less often chose DS for stress, anxiety, depression, or sleeping problems than students declaring a high knowledge (10.7% and 29.6% vs. 46.6%, respectively). Detailed comparisons between subgroups of respondents in the usage of DS for stress, anxiety, depression, or sleeping problems are presented in Table 2.

**Figure 4:**
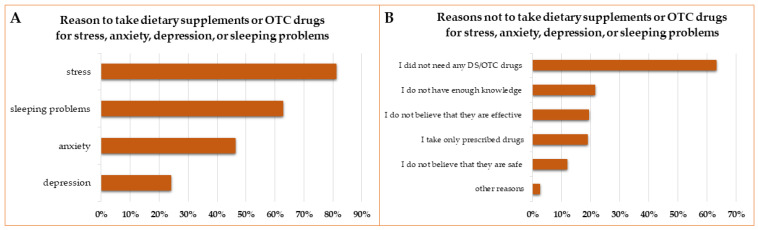
The *reasons for choosing* DS or OTC drugs for stress, anxiety, depression, or sleeping problems (**A**), the reasons not to take DS or OTC drugs for stress, anxiety, depression, or sleeping problems; some other reasons for not using DS or OTC drugs are mentioned in the main text (**B**).

Stress was the most common reason for taking this kind of DS, followed by sleeping problems (Figure 4A). Students not taking the DS for stress, anxiety, depression, or sleeping problems in most cases did not need them (63.2%). The second reason was insufficient knowledge about these kinds of DS and it was declared by 21.4% of respondents. It is worth noting that every 5th student did not believe that DS are effective, and every 10th student did not believe that they are safe. A few persons mentioned coping with stress by engaging in hobbies, sports, long walks, or leisure as better and safer than using any tablets (Figure 4B).

**Figure 5:**
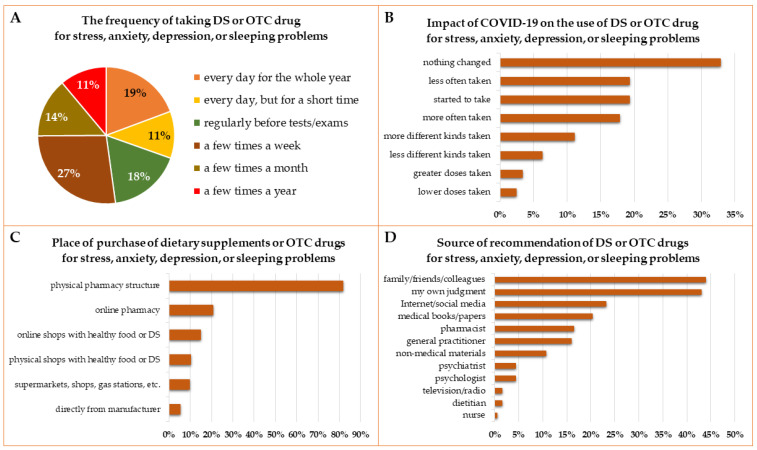
The frequency of taking DS and OTC drugs (**A**), the impact of the COVID-19 pandemic on the pattern of their use (**B**); place of purchase (**C**), and source of recommendation (**D**) of dietary supplements or OTC drugs for stress, anxiety, depression, or sleeping problems.

No specific pattern of the use of DS or OTC drugs for stress, anxiety, depression, or sleeping problems was noted, but the largest group of the students (27%) took these products a few times a week (women constitute 85% of this group), followed by regularly, every day, and regularly but only before stressful situations (Figure 5A). About 33% of students declared that the COVID-19 pandemic did not change the pattern of usage of DS for stress, anxiety, depression, or sleeping problems. Nineteen percent of students took DS or OTC drugs less often than before the pandemic and the same number of persons started to take them during the pandemic. Among students who started taking DS or OTS drugs during the pandemic, 82% were women, which is a slightly higher ratio than in the overall analyzed population (74%). Slightly less (18%) took DS for stress, anxiety, depression, or sleeping problems more often, and 11% took more different kinds of them (Figure 5B).

Over 80% of students bought DS or OTC drugs for stress, anxiety, depression, or sleeping problems in pharmacies (Figure 5C). The second place was online pharmacies (21%). Online or brick-and-mortar shops with healthy food were also popular (15% and 10%, respectively). Most students took DS or OTC drugs after recommendation by family members, friends, or colleagues (44%), or based on their own judgment (43%). Other sources of recommendation were the Internet and social media and medical books and research papers (Figure 5D).

**Figure 6:**
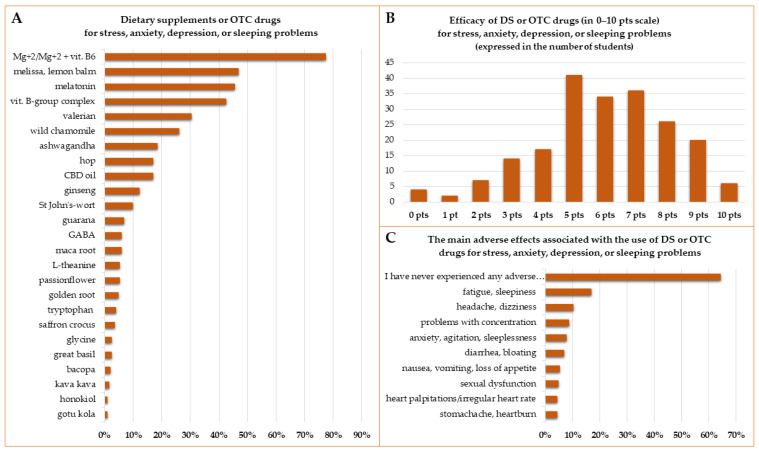
The most commonly chosen dietary supplements or OTC drugs for stress, anxiety, depression, or sleeping problems in the academic year 2020/2021. CBD—cannabidiol (**A**), the efficacy of DS or OTC drugs assessed by the students (0—completely ineffective, no improvement, 10—fully effective, complete disappearance of symptoms) (**B**), and the main adverse effects combined with the use of dietary supplements or OTC drugs for stress, anxiety, depression, or sleeping problems (**C**). Figure 6C presents adverse effects which were indicated by more than 4% of respondents; less commonly reported adverse effects are mentioned in the main text. The genus and species names of herbs from the Figure 6A are as follows (in the order from the most frequently used): *Melissa officinalis* L. (melissa, lemon balm), *Valeriana officinalis* L. (valerian), *Matricaria* recutita L. (wild chamomile), Withania *somnifera* L. (ashwagandha), *Humulus lupulus* L. (hop), *Panax ginseng* C.A. Mey. (ginseng), *Hypericum perforatum* L. (St. John’s wort), *Paullinia cupana* Kunth (guarana), *Lepidium peruvianum* Chacon (maca), *Passiflora incarnata* L. (passionflower), *Rhodiola rosea* L. (golden root), *Crocus sativus* L. (saffron crocus), *Ocimum basilicum* L. (great basil), *Bacopa monnieri* L. (bacopa), *Piper methysticum* G. Forst. (kava kava), *Magnolia officinalis* Rehd. et Wils. (honokiol), and *Centella asiatica* L. (gotu kola).

The DS or OTC drugs most frequently chosen (by 77% of students) for stress, anxiety, depression, or sleeping problems were magnesium or a combination of magnesium with vitamin B6. *Melissa officinalis* L. (melissa, lemon balm), melatonin, or a Vitamin-B group complex was taken by 40–50% of students who decided to use DS or OTC drugs for the aforementioned problems. *Valeriana officinalis* L. root (valerian root) was used by 30% and *Matricaria recutita* L. (wild chamomile) was used by 26%. *Withania somnifera* L. (ashwagandha), *Humulus lupulus* L. (hop), or cannabidiol (CBD) oil was taken by more than 17%, but less than 20% of students were taking these DS for stress, anxiety, depression, or sleeping problems. Except for ginseng (12%), the use of other DS was declared by less than 10% of students (Figure 6A). No significant differences between subgroups of respondents were found in the number of DS taken for stress, anxiety, depression, or sleeping problems, with the exception that students taking at least three different DS for the indications mentioned earlier took other DS less often than students taking less than three DS for stress, anxiety, depression, or sleeping problems (27% vs. 46%, *p* = 0.012). In the subgroup of respondents taking DS for stress, anxiety, depression, or sleeping problems, herbs were more often chosen by Polish Division students than ED students (88% vs. 63%, *p* = 0.0000), women than men (85% vs. 67%, *p* = 0.0098), and by students taking at least three different DS for the aforementioned problems (84% vs. 16%, *p* = 0.0000). Only slightly more than 9% of men chose professional healthcare providers as a source of knowledge about the DS, whereas 40% of women did so (*p* = 0.0001). In addition, students in later years of their studies used the advice of healthcare professionals about DS more often (38% for clinical vs. 22% for preclinical students, *p* = 0.0427).

On a 0–10-point scale (0—completely ineffective, 10—fully effective), most students assessed the efficacy of taken DS or OTC drugs as moderate (44%) or high (43%), however, very high efficacy (10 points) was indicated by less than 3% of respondents who used DS for stress. Most often (19%) students assessed efficacy at five points (Figure 6B). No significant differences were found in the assessed efficacy of DS or OTC drugs for stress, anxiety, depression, or sleeping problems between different subgroups of respondents. DS or OTC drugs for stress, anxiety, depression, or sleeping problems were well tolerated and 64% of students who used them did not declare any adverse effects during their use. Fatigue or sleepiness was reported by 17% of students. Problems related to the central nervous system (anxiety, agitation, sleeplessness, problems with concentration, headache, dizziness, fatigue, and sleepiness) were reported most often. All gastrointestinal adverse effects together (nausea, vomiting, loss of appetite, diarrhea, bloating, constipation, stomachache, or heartburn) were noted 39 times, which constitutes almost 22% of all the mentioned adverse events. Other adverse effects were observed less frequent (Figure 6C). Besides the adverse effects mentioned in Figure 6C some, students reported constipation, too low or too high blood pressure, problems with breathing or asthma attacks, itching, rash, urticaria, deterioration of laboratory tests results, and paresthesia.

For stress, anxiety, depression, or sleeping problems, 16% of students used POM, either regularly or only sometimes, in the periods of increased stress such as semester tests or exams (Figure 3D). Significant differences were found between subgroups of respondents who never took POM and those who took them (regularly or sporadically). More Polish Division (19.5%) than English Division (9.2%) students used POM (for the whole academic year the ratio was 11.1% vs. 3.7% for PL and ED students, respectively). Significant differences in taking POM were also found between men and women. More women (19.1%) than men (7.95%) used POM (for those taking them regularly during the whole academic year the ratio was 11.4% vs. 3.8% and for those taking them sporadically—8.2% vs. 3.8%, for women and men, respectively). Besides the impact of a high level of stress on the decision about the use of POM (regularly or sporadically), students who declared an unhealthy diet, higher level of knowledge about DS, and taking DS or OTC drugs for stress, anxiety, depression, or sleeping problems more often decided to use POM. Detailed comparisons are presented in Table 3.

### 3.4. The Use of Other Dietary Supplements and OTC Drugs

**Figure 7:**
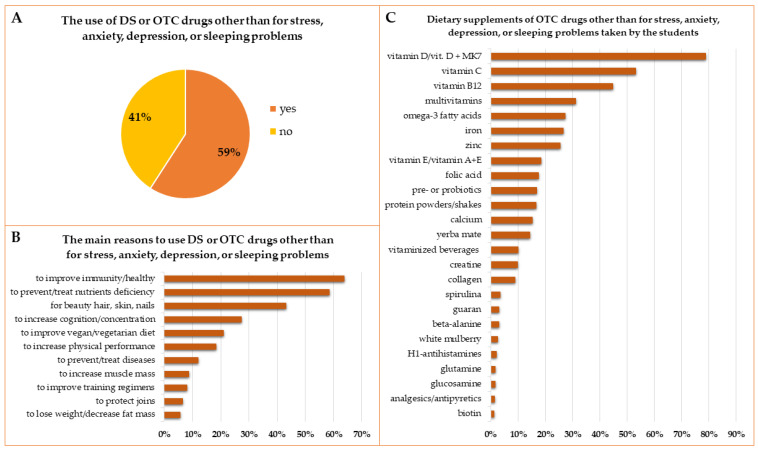
The general use of dietary supplements or OTC drugs other than for stress, anxiety, depression, or sleeping problems in the academic year 2020/2021 (**A**), the main reasons to take this kind of DS or OTC drugs (**B**), and DS or OTC drugs other than for stress, anxiety, depression, or sleeping problems that were the most commonly chosen by the students (MK7—menaquinone-7) (**C**). The genus and species names of herbs from Figure 7C are as follows (in the order from the most frequently used): *Ilex paraguariensis* A.St.-Hil. (yerba mate), *Paullinia cupana* Kunth (guarana), and *Morus alba* L. (white mullberry).

Fifty-nine percent of respondents declared the use of DS or OTC drugs other than for stress, anxiety, depression, or sleeping problems (Figure 7A). Gender has been reported to influence this decision (63% of women took DS and only 49% of men, *p* = 0.0023). However, the most important differences were found between English and Polish Division students (*p* = 0.0000). Only 38% of English Division students declared the use of other DS or OTC drugs, whereas 68% of Polish Division students took DS or OTC drugs other than for stress, anxiety, depression, or sleeping problems in the last academic year. Significant differences were observed also between the main Faculties. Students of the Medical Faculty were almost equally divided into users and nonusers of DS or OTC drugs, and the share of students of the Dentistry Faculty who used DS or OTC drugs was 55%. Other trends were described in the Health Science Faculty, where 61% of students used the products mentioned above, and in the Pharmacy Faculty, where 76% of students took at least one DS or OTC drug. Differences in the usage of DS or OTC drugs depended additionally on the declared type of diet. Students declaring definitely healthy, rather healthy, or students who did not define their diet more often used dietary supplements or OTC drugs (64%, 64%, and 65%, respectively) than students declaring a rather unhealthy or definitely unhealthy diet (53.5% and 43%, respectively). Students who rated their knowledge about DS as high or moderate more often chose dietary supplements than students who rated their knowledge as low. The detailed comparisons in subgroups of respondents are presented in Table 4.

Most of the students choosing DS or OTC drugs indicated (Figure 7B) the improvement of immunity and willingness to stay healthy (64%) and prevention or treatment of a variety of microelement or nutrient deficiencies (54%) as the main reasons. Other reasons for taking medications were the desire to have beautiful hair, skin, and nails (43%), to increase cognition, concentration, or alertness (27%), and to complement a vegan/vegetarian diet (21%). Some less frequent reasons, not listed in Figure 7B, included supplementation of the medical elimination diet, improvement of sexual performance, headache or migraine, protection or improvement of liver function, and gastrointestinal health. Students also declared the use of OTC antihistamines and analgesic, antipyretic, or anti-inflammatory drugs.

The most often chosen dietary supplements or OTC drugs other than for stress, anxiety, depression, or sleeping problems were vitamin D, with or without menaquinone-7 (MK7) (79%), vitamin C (53%), vitamin B12 (45%), multivitamin preparations (31%), zinc (25%), iron (27%), and omega-3 fatty acids (27%). Some other well-known supplements were chosen by 10% to 20% of the users, and many by less than 10% of students (Figure 7C).

### 3.5. Knowledge of Dietary Supplements 

**Figure 8:**
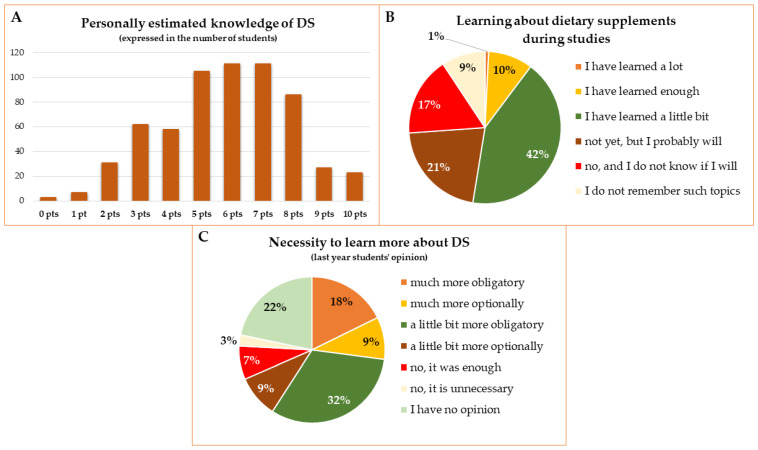
The level of knowledge about DS self-assessed by the students (0—I do not know anything, 10—I think I know very much) (**A**), the information given by students about topics concerning dietary supplements (**B**), and opinion of final-year students about the necessity to learn more about dietary supplements during the studies at the Wroclaw Medical University (**C**).

Most of the students rated their level of knowledge about dietary supplements as moderate or high, from 5 to 8 points on a 0-10-point scale (0—I do not know anything, 10—I think I know very much). Two hundred and sixteen persons rated it at 5–6 points and 197 students at 7–8 points (which corresponds to 34.6% and 31.6% of respondents, respectively). Only 1.6% assessed their knowledge as very low (0–1 point) and 8% as very high (9–10 points) (Figure 8A). For statistical analysis, 0–3 points were considered as low, 4–6 points were considered moderate, and 7–10 points were considered a high level of knowledge about dietary supplements. As described previously, the level of knowledge influenced the decisions about DS usage and choices of the type of diet. Students who rated their knowledge as low used different types of DS less often. More students with a high level of knowledge assessed their diet as definitely healthy (52.5%) compared to students declaring a definitely unhealthy diet (31%). The details concerning the mean levels of self-assessed knowledge about DS in the subgroups of students is presented in Supplementary Table S1.

Only 11% of the respondents claimed that during their studies they had learned a lot or enough about DS and 21% believed that they would learn about such topics during the courses in the future (Figure 8B). Among final-year students, 7% considered that there was enough information about dietary supplements provided in the program of their courses and merely 3% of respondents considered it unnecessary to learn more about dietary supplements. According to the majority of respondents (68%), students should learn more about DS in compulsory classes, while 18% believe that these topics should be discussed in optional classes (Figure 8C).

### 3.6. Associations between Different Factors and the Use of DS or POM 

The logistic regression analysis results with an odds ratio (OD), *B* coefficient, and confidence interval (CI) values are presented in detail in Table 5. The most important associations were as follows: (1) English Division students were less likely than Polish Division students to take any dietary supplements, other than for stress, anxiety, depression, or sleeping problems, and POM for stress, anxiety, depression, or sleeping problems; (2) compared with their female counterpart, men took other DS for stress and POM for stress less often; (3) students on higher years of medical education (5th and 6th) were much more willing to take DS other than for stress, anxiety, depression, or sleeping problems, as well as POM for stress when compared to the students who had just started their education at the university (2nd-year students); (4) persons declaring an unhealthy diet or who were uncertain about their diet type were more likely to use DS for stress or POM for stress; (5) the high self-estimated stress level impacted the use of DS for stress or POM for stress; (6) an increased level of declared knowledge about the DS increases the overall use of DS, DS for stress, and DS used for other reasons than for stress and POM for stress, however, the latter increases insignificantly.

## 4. Discussion

As of October 23, 2021, there were 4914 students from the second to final year of their majors enrolled with the Wroclaw Medical University (data from the Dean Offices). Students of the Medical Faculty constituted 51% of this number, compared to students of the Dentistry Faculty—8%, the Pharmacy Faculty—21%, and the Health Science Faculty—19%. In the survey conducted by the authors, this proportion was similar in the case of the Medical and Dentistry Faculties (51% and 9% of respondents), and a slightly higher number of students from the Pharmacy Faculty responded with a lower response rate from the Health Science Faculty students (30% and 10%, respectively). The lower than expected response rate from the Heath Science Faculty may be explained by the fact that students of this Faculty do not have any classes in our Department of Pharmacology, and they do not know the authors of the questionnaire, which could have influenced their willingness to take part in the study. Greater differences were seen between separate majors of studies, and a very low response from students of public health or nursing is very surprising and difficult to explain. The best response was noticed for Medical and Dentistry majors of the English Division (29% and 24%, respectively) which may be explained by the involvement of English Division students in the organization of this study. Similarly, the greatest group of respondents was from the 3rd year, which may be explained by the fact that these students are currently attending the Pharmacology and toxicology course (medical students) and Pharmacology course (dentistry students) in our department. For example, in the study of Orayj et al. [19], most of the respondents were students in their fourth or final year. Females predominated in our study, but it is consistent with the available data that, at the Wroclaw Medical University, there are about 2.5 times more female than male students [24]. In some other studies, women also predominate among respondents when surveyed about lifestyle, DS or OTC drug use [9,11,12,19], but there was no mention of the men to women ratio in the analyzed population. However, a Danish study indicated that men and women are similarly willing to participate in surveys except for “paper and pencil mail surveys”, in which women are more likely to take part [25].

Time at university is a very special time in the life of young people preparing for adulthood. Many factors may significantly influence their diet during their studies and impact their future habits. Some of these factors may be, for example, greater autonomy from parents, limited funds for daily needs, the influence of new colleagues from different countries and cultures, stress, a lot of learning and no time to prepare healthy and wholesome meals, or little time for rest and physical activity [26]. In the presented study, the majority of students (55%) considered their diet healthy and less of them (39%) unhealthy. A similar number of students defined their diet as definitely healthy (61 persons) or definitely unhealthy (58 persons). The results of studies conducted at veterinary schools in Canada and the Unites States were similar. About 58% of veterinary students assessed their diet as moderately healthy and, at the same time, over 90% of respondents perceived a “busy lifestyle” as the problem to change dietary habits [27]. However, to objectively assess if the diets of Wroclaw Medical University students are actually healthy, a survey focused on this aspect would be needed.

In the study of Belogianni et al. [28], it was found that almost 47% of surveyed students had a good level of knowledge about nutrition, especially regarding healthy food choices, while their level of knowledge about the food sources of nutrients was lower. In the presented study, the majority of students also declared that their diet is healthy, which may confirm that young people take care of their food choices. However, it is worth mentioning that in addition to 39% of students who perceived their diet as unhealthy, the next 6% of our respondents were unable to state whether their diet was healthy, balanced, and sufficient or not. It is suggested that this insecurity may be associated with dietary choices among students who less often consume healthy food [29]. It indicates the need for good dietary programs and education from an early stage of medical studies irrespective of the major. As it was found in the study of Coleman et al. [30], education in the first year of medical studies changed their dietary choices for the better and it may also impact the possibility to advise patients on nutrition in the future. A recent review [31] also showed that a variety of teaching approaches provided students with many benefits which are particularly important since an unhealthy diet is the cause of about 20% of deaths.

In many countries, the COVID-19 pandemic impeded access to family doctors and specialists. Dissatisfaction with telemedicine caused patients to search for advice in pharmacies or, what was especially worrisome, on the Internet [6,17]. DS or OTC drugs are available without prescription and may be easily bought on the Internet and delivered directly to the home. Self-medication is a well-known problem that increased during the situation of the COVID-19 pandemic. It concerns different groups of patients including students at medical universities [32]. However, the tendency toward self-medication among students at medical universities was observed also before the time of the pandemic. Students most often used OTC drugs to relieve fever, pain, or abdominal problems [18]. Some data indicated that even medical students practicing self-medication did not always have the knowledge of the possible adverse effects and very rarely had any medical tests or consultations before self-treatment [33]. Other problems are with the use of outdated OTC medications or the use of doses higher than recommended, even among medical or pharmacy students [18]. 

The WHO estimates that almost 450 million people all over the world suffer from stress-related disorders [34]. Independently from the COVID-19 pandemic, exams are the main sources of stress for students at Medical Universities, and test anxiety may affect about 20–40% of students [35]. It was found that during the exams, students often suffer from headaches, which were one of the most common reasons to take OTC drugs, such as analgesics [19]. The analysis of the answers obtained in our survey shows that most of the students (72%) of Wroclaw Medical University rated their stress level as high (7–8 points) or very high (9–10 points). In the presented study, a similar percentage of students in different Divisions or Faculties declared high levels of stress in the last academic year, suggesting that they were exposed to similar stressors. However, Pharmacy Faculty students assessed their stress as significantly higher than Medical Faculty students did. This may be connected to the specific nature of this program, requiring memorizing a lot of information. In a study performed in the United Kingdom, it was found, using an objective scale, that the high level of stress among the undergraduate pharmacy students were comparable to the level of stress experienced by pharmacy students in the United States, but greater than in the general population [36].

One-third of the respondents used DS or OTC drugs for stress, anxiety, depression, or sleeping problems during the last academic year (2020/2021). No significant influence of gender on the decision to take DS was observed, but women used DS or OTC drugs for stress, anxiety, depression, or sleeping problems slightly more often than men (35.4% vs. 27.4%). This is difficult to compare with other studies because many of them focus on the general use of dietary supplements rather than specifically on supplements for stress, anxiety, depression, or sleeping problems; but in general, the trend is that women use DS more often [37,38]. Among the many reasons for taking the DS or OTC drugs reported by the students or young adults are relief of fatigue, focus enhancement [10], stress management, mental focus, concentration, improving the duration and quality of sleep [6], or memory enhancement [39,40,41]. 

A high level of stress impacted the use of POM among respondents in the presented study. In general, it is not surprising, but data about the use of antidepressants or benzodiazepines during the COVID-19 pandemic are inconsistent. Despite the increased rate of depressive and anxiety disorders in Canada during the COVID-19 pandemic, the prescribing of POM was not increased, or even was transiently, but was significantly lower at the beginning of the pandemic [23]. Similar trends were noted by Fornari et al. [42] when the use of antidepressants in Italy was lower during the lockdown period than before the pandemic, along with the increase in the interruption of treatment despite the increased prevalence of depressive and post-traumatic stress disorders, especially in young people and students [43]. On the other hand, in the United States or the United Kingdom, an increase in the number of prescriptions for depression, anxiety, and insomnia was noticed, especially in the first few months of the COVID-19 pandemic [44]. The present authors do not know how exactly the pattern of the use of POM for stress, anxiety, depression, or sleeping problems changed in the group of our respondents in this study, but it may be an interesting issue for analysis. 

In the present study, stress and sleeping problems were the most common reasons for the use of DS or OTC drugs. This was reflected in the choosing of magnesium salts with or without vitamin B6, melissa, melatonin, or valerian. In the second PLifeCOVID-19 Online Study, 25% of participants used DS due to stress and 13% because of sleeping problems [6]. In an analysis of the group of all respondents in the present study (not only the subgroup taking DS for stress, anxiety, depression, or sleeping problems), stress was indicated as a reason to use dietary supplements by 27%, which is similar to data from a paper of Hamulka et al. [6]. Sleeping problems were the reason for supplementation with DS in 21% of our respondents in the present study, which is slightly more often than in the previously cited paper. It may be explained by the fact that in the academic year 2020/2021, the students in the study presented in this paper spent a lot of time in online classes, seminars, and lectures, which might have influenced their sleep patterns. It is known that exposure to artificial light, including light from the computer screen, may lead to insomnia [45].

The study did not find any specific impact of the COVID-19 pandemic on the frequency or pattern of the use of DS or OTC drugs for stress, anxiety, depression, or sleeping problems, and most students declared that nothing changed in the use of these kinds of supplements during the last academic year. Most students used them a few times a week and less of them every day. Daily or weekly use was also reported by other authors [41,46]. However, in the study by Hanna et al. [47], pharmacy students most often took the OTC medication once a month, but the survey was performed before the COVID-19 pandemic and mostly concerned drugs available without prescription, not the products classified as DS. It is worth mentioning, that the greatest increase in the use of DS was observed in the first wave of the COVID-19 pandemic [6], and the study presented in this article covers the mid-period of the current pandemic (from October 2020 to September 2021). However, 19% of persons who used DS for stress, anxiety, depression, or sleeping problems started to take them during the pandemic time. An analysis of the results of the first and the second PLifeCOVID-19 Study indicated that in the 1st and 2nd study, 4.4% and 9.3% of respondents, respectively, started using all the DS during the pandemic, and 9.3% and 25% started using some of the DS in that time [6]. Karbownik et al. [48] analyzed medication- and DS-related behaviors during the lockdown and did not find any significant differences except for the stronger belief of the respondents that quality, efficacy, safety, and composition of DS are well controlled, and the lower accessibility of DS advertisements. 

Respondents in the presented survey most often bought DS or OTC drugs for stress, anxiety, depression, or sleeping problems in brick-and-mortar pharmacies or online pharmacies, and the most important sources of recommendation were family/friends/colleagues, own judgment, and the Internet/social media. It was very similar to other papers describing the general use of DS or OTC drugs [6,37,38,46]. It is important that in some studies, both users and non-users of DS considered media as a very important factor influencing the decisions about the use of DS [13]. It is very promising that students bought DS in pharmacies, but of greater concern is the source of recommendation. More emphasis should be placed on doctor’s or pharmacist’s opinion when choosing dietary supplements or over-the-counter medications. In the presented study, general practitioners or pharmacists were the sources of recommendation for only 16% of persons taking DS or OTC drugs for stress, anxiety, depression, or sleeping problems, which is much less than in some other studies [39].

Most of the students surveyed by the authors did not experience any adverse effects during the use of DS, but many reported fatigue or sleepiness, headaches or dizziness, and a variety of gastrointestinal problems. These symptoms, however, are difficult to distinguish from symptoms of work overload, stress, or even depression [49]. A very similar profile of adverse effects of dietary supplements was reported by Samreen et al. [41], Kobayashi et al. [37], Jahan et al. [40], and Alqrache et al. [10]. It is worth mentioning that in choosing medical products available without prescription, almost 80% of pharmacy students consider safety as important in recommending the product to others and the third most important factor when they chose the OTC drug for themselves [47].

The use of DS or OTC drugs other than for stress, anxiety, depression, or sleeping problems was reported by 59% of respondents, but considering both types of DS, supplementation was declared by 436 persons which constitutes 70% of students who answered the questionnaire. It was found that there are great differences between countries in the use of dietary supplements in young people and children, ranging from 20% in Australia or Japan to 35–37% in Italy or the USA [50]. However, studies performed in groups of students indicated a greater usage of DS, e.g., 52% in the USA, 54% in Australia, and 68% in Serbia [38,51,52,53]. In the present study, the greatest differences in the prevalence of other DS use were observed between the Polish and English Divisions. Among English Division students, 38% declared the use of other DS or OTC drugs, which is consistent with results from other countries [50,53], but in the Polish Division, 68% of students took DS, which is more similar to the data obtained by Miljković et al. [38] or in a study by Brodziak et al. [54]. It is, however, worth mentioning that many studies were performed before the COVID-19 pandemic. In the paper of Yang published in April 2020 [55], the prevalence of the intake of dietary supplements in college students was also high and was declared by 72.4% of participants. The differences in the use of DS observed in our study, on the one hand, may be explained by the different access to the advertisements of DS between English and Polish Division students. The latter may see the advertisements in many different sources in the national language whereas the English Division students may have some problems understanding them in Polish. On the other hand, the authors found that the use of DS for stress, anxiety, depression, or sleeping problems was similar in both Divisions, so access to the information is for sure not the only factor responsible for such differences in the use of DS other than for stress, anxiety, depression, or sleeping problems.

At the Wroclaw Medical University, participants indicated improvement of immunity and health and the prevention or treatment of nutrient deficiencies as two main reasons to use other dietary supplements or OTC drugs. Similar reasons for supplementation were mentioned by Hamulka et al. [6] or by Žeželj et al. [53]. This is confirmed by the choices made by the students of the Wroclaw Medical University with frequent supplementation with DS or OTC drugs with vitamin D, vitamin C, vitamin B12, multivitamins, omega-3 fatty acids, or zinc. 

The surveyed students most often declared a moderate to high level of knowledge about dietary supplements. This subsequently influenced the dietary choices and the decisions about the use of dietary supplements. A comparison of reasons for the use of dietary supplements and most-often chosen DS indicates that students know what DS may give specific benefits, as later confirmed in the assessment of the efficacy of the used DS or OTC drugs made by the students who used them. As described in some studies, the efficacy of the product available without prescription is the first factor influencing the choice of OTC medication for personal use by the pharmacy students and the second most important reason (after safety) for making decisions about OTC drugs recommended to patients [47]. In the area of DS for stress, anxiety, depression, or sleeping problems, the most common reason to use the DS or OTC drugs was stress, followed by sleeping problems. The DS or OTC drugs taken most often were magnesium (with or without vitamin B6), melissa, melatonin, a vitamin B complex, valerian, chamomile, and ashwagandha. In the group of students taking other dietary supplements, the need to increase their immune defense and the supplementation of nutrient deficiency were the two most important reasons to take DS or OTC drugs with vitamins D, C, B12, multivitamins, omega-3 fatty acids, or zinc, chosen by 25% (zinc) to 79% (vitamin D) of DS users. The presented study has a limitation in this regard because the survey did not contain any specific questions checking the respondents’ knowledge. Karbownik et al. [56] surveyed medical and non-medical participants with specific questions about DS and concluded that the level of knowledge on DS is low and in contrast with data available on self-assessed familiarity with dietary supplements, which is often overestimated. 

Another aspect of knowledge on DS is the awareness of the potential adverse effects or the risk of an overdose of some DS or OTC drugs. Some studies indicate that many students consider DS as safe, without the risk of harm. It depends on the major of the studies and may differ significantly. In some surveys, only 25% of students indicated that DS may be harmful to health [10], but in some others, about 87% were aware of some risk to health [41]. In the study by Bekele et al., almost 25% of participants rarely or never read the patient information leaflet, and over 20% rarely or never checked the expiry date even though they were medical or pharmacy students [18]. The present authors did not directly assess the level of knowledge in the context of adverse effects or DS–drug interactions, but in the group of students who did not decide to use DS for stress, anxiety, depression, or sleeping problems, 12% did not believe that they are safe and 21% assessed their knowledge as insufficient. It suggests the need for education in this field regardless of the major of studies.

Only 1% of students declared that they have learned a lot about DS during their studies, but 52% stated that they learned enough or at least acquired a very limited amount of knowledge. Final-year students were asked about their opinion on the necessity to introduce classes on dietary supplements and 68% of them expressed the opinion that during the studies at Wroclaw Medical University, there should be more about dietary supplements (a little bit or much more), and 50% of students thought that it should be provided as an obligatory course. In this group, 46% were medical and 28% were pharmacy students. In the study by Axon et al. [51], pharmacy students considered knowledge about dietary or herbal supplements as important, but they indicated that their knowledge was limited, which led to the conclusion that good education is necessary from the early years of medical majors. In general, it is stated that nutritional education is under-represented and underestimated in medical schools, and many medical school students reported inadequate knowledge and teaching methods that require employing more modern, interactive, and student-engaging approaches [31]. Different methods may be used to improve the students’ knowledge about DS or OTC drugs, especially in the experiential learning formulas, such as in-class activities (simulated patients) or work-based placements [57].

## 5. Limitations

The main limitation of the study was the impossibility to survey graduates. The survey (link) was distributed to students by the representatives of each year using the University email addresses and graduates no longer have such emails. Additionally, the study was conducted only at Wroclaw Medical University, and expanding the study to include students from other universities would certainly be worthwhile.

## 6. Conclusions

The results of the survey presented in this article revealed that 70% of students of the Wroclaw Medical University declare the use of any DS or OTC drugs. They are more often chosen by Polish Division students, women, Pharmacy students, persons studying at higher years, declaring a healthy diet, a high level of knowledge about DS, and a high level of stress. The most important factors influencing the decision to take any kind of DS were Division, year of the studies, declared type of diet, self-assessed level of stress, and level of knowledge about DS. It is noteworthy that 72% of respondents assessed their stress level as high, and over 30% of respondents were taking DS or OTC drugs for stress, anxiety, depression, or sleeping problems. A high level of stress and high knowledge about DS were significant factors influencing the decision to use DS or OTC drugs for stress, anxiety, depression, or sleeping problems in this group of students. Although most students rated their level of knowledge about DS as moderate to high, in the opinion of 50% of final-year students, they should obligatorily learn slightly more or much more about DS during their studies. Since many factors can significantly affect the quality of life, it can be concluded that students take various DS or OTC drugs to improve their quality of life, e.g., by reducing stress or improving immunity.

Overall, the results of this study may also be helpful to plan more detailed studies on students’ QoL, reasons for stress, or objective knowledge about DS or OTC drugs. This may further help to identify underlying problems and take specific actions at the Wroclaw Medical University to help students to cope with stress and to make informed choices concerning DS, as well as allow them to improve their knowledge about DS in modern, active, and interesting formulas so that they can reliably advise patients in the future.

## Figures and Tables

**Table 1:** The overall use of dietary supplements among Wroclaw Medical University Students in the academic year 2020/2021. Analyses were performed using the chi-square test.

The Overall Use of Dietary Supplements				
		YES	NO	*p*-Value
**Division**	Polish (*n* = 434)	77.19%	22.81%	*p* = 0.0000
	English (*n* = 190)	53.16%	46.84%	
**Gender**	Women (*n* = 463)	72.79%	27.21%	*p* = 0.0142
	Men (*n* = 157)	62.42%	37.58%	
**Faculty**	Medical (*n* = 320)	61.56%	38.44%	*p* = 0.0000
	Pharmacy (*n* = 189)	82.01%	17.99%	
	Dentistry (*n* = 53)	71.7%	28.3%	
	Health Science (*n* = 62)	74.19%	25.81%	
**Year of Studies**	2nd (*n* = 166)	65.06%	34.94%	*p* = 0.0609
	3rd (*n* = 215)	67.44%	32.56%	
	4th (*n* = 116)	69.83%	30.17%	
	5th (*n* = 61)	80.33%	19.67%	
	6th (*n* = 66)	80.3%	19.7%	
**Level of Studies**	pre-clinical (*n* = 497)	67.20%	32.80%	*p* = 0.0041
	clinical (*n* = 127)	80.31%	19.69%	
**Diet**	healthy diet (*n* = 341)	73.61%	26.39%	*p* = 0.0256
	unhealthy diet/difficult to say (*n* = 283)	65.37%	34.63%	
**The Self-Estimated Stress Level** **(0–10 pts Scale)**	high, >6 pts (*n* = 449)	72.61%	27.39%	*p* = 0.0098
	moderate, 4–6 pts (*n* = 139)	66.19%	33.81%	
	low, <4 pts (*n* = 36)	50%	50%	
**The Self-Estimated Knowledge about DS** **(0–10 pts Scale)**	high, >6 pts (*n*= 247)	83.4%	16.6%	*p* = 0.0000
	moderate, 4–6 pts (*n* = 274)	71.17%	28.83%	
	low, <4 pts (*n* = 103)	33.98%	66.02%	

**Table 2:** The use of dietary supplements for stress, anxiety, depression, or sleeping problems among Wroclaw Medical University Students in the academic year 2020/2021. Analyses were performed using the chi-square test.

The Use of Dietary Supplements for Stress, Anxiety, Depression, or Sleeping Problems				
		YES	NO	*p*-Value
**Division**	Polish (*n* = 434)	34.56%	65.4%	*p* = 0.2653
	English (*n* = 190)	30%	70%	
**Gender**	Women (*n* = 463)	35.42%	64.58%	*p* = 0.0651
	Men (*n* = 157)	27.39%	72.61%	
**Faculty**	Medical (*n* = 320)	31.25%	68.75%	*p* = 0.7735
	Pharmacy (*n* = 189)	34.92%	65.08%	
	Dentistry (*n* = 53)	35.85%	64.15%	
	Health Science (*n* = 62)	34.48%	64.52%	
**Year of studies**	2nd (*n* = 166)	28.92%	71.08%	*p* = 0.2059
	3rd (*n* = 215)	33.49%	66.51%	
	4th (*n* = 116)	31.90%	68.10%	
	5th (*n* = 61)	45.90%	54.10%	
	6th (*n* = 66)	33.33%	66.67%	
**Level of studies**	pre-clinical (*n* = 497)	31.59%	68.41%	*p* = 0.0965
	clinical (*n* = 127)	39.37%	60.63%	
**Diet**	healthy diet (*n* = 341)	29.91%	70.09%	*p* = 0.0575
	unhealthy diet/difficult to say (*n* = 283)	37.10%	62.70%	
**The self-estimated stress level** **(0–10 pts scale)**	high, >6 pts (*n* = 449)	37.64%	62.36%	*p* = 0.0006
	moderate, 4–6 pts (*n* = 139)	23.02%	76.98%	
	low, <4 pts (*n* = 36)	16.67%	83.33%	
**The self-estimated knowledge about DS** **(0–10 pts scale)**	high, >6 pts (*n*= 247)	46.56%	53.44%	*p* = 0.0000
	moderate, 4–6 pts (*n* = 274)	29.56%	70.44%	
	low, <4 pts (*n* = 103)	10.68%	89.32%	

**Table 3:** The use of prescription-only medicines (POM) for stress, anxiety, depression, or sleeping problems among Wroclaw Medical University Students in the academic year 2020/2021. Students who marked the answer “prefer not to disclose” are not included in the statistical analysis. Analyses were performed using the chi-square test.

The Use of Prescription-Only Medicines (POM) for Stress, Anxiety, Depression, or Sleeping Problems				
		YES, Regularly or Sometimes	NO, Never	*p*-Value
**Division**	Polish (*n* = 421)	19.48%	80.52%	*p* = 0.0017
	English (*n* = 184)	9.24%	90.76%	
**Gender**	Women (*n* = 450)	19.11%	80.89%	*p* = 0.0013
	Men (*n* = 151)	7.95%	92.05%	
**Faculty**	Medical (*n* = 313)	15.97%	84.03%	*p* = 0.3688
	Pharmacy (*n* = 184)	36.36%	80.43%	
	Dentistry (*n* = 50)	14%	86%	
	Health Science (*n* = 58)	10.34%	89.66%	
**Year of studies**	2nd (*n* = 158)	10.76%	89.24%	*p* = 0.0997
	3rd (*n* = 209)	19.62%	80.38%	
	4th (*n* = 113)	14.16%	85.84%	
	5th (*n* = 60)	16.67%	83.33%	
	6th (*n* = 65)	23.08%	76.92%	
**Level of studies**	pre-clinical (*n* = 480)	15.42%	84.58%	*p* = 0.2173
	clinical (*n* = 125)	20%	80%	
**Diet**	healthy (*n* = 336)	13.1%	86.9%	*p* = 0.0152
	unhealthy/difficult to say (*n* = 269)	20.45%	79.55%	
**The self-estimated stress level** **(0–10 pts scale)**	high, >6 pts (*n* = 431)	19.26%	80.74%	*p* = 0.0101
	moderate, 4–6 pts (*n* = 138)	9.42%	90.58%	
	low, <4 pts (*n* = 36)	8.33%	91.67%	
**The self-estimated knowledge about DS** **(0–10 pts scale)**	high, >6 pts (*n* = 241)	23.06%	76.35%	*p* = 0.0001
	moderate, 4–6 pts (*n* = 264)	13.64%	86.36%	
	low, <4 pts (*n* = 100)	6%	94%	
**DS for stress/anxiety/depression/sleeplesness**	users (*n* = 197)	30.46%	65.54%	*p* = 0.0000
	non-users (*n* = 408)	9.56%	90.44%	
**Other DS**	users (*n* = 358)	18.99%	81.01%	*p* = 0.0352
	non-users (*n* = 247)	12.55%	87.45%	
**Any DS**	users (*n* = 422)	19.67%	80.33%	*p* = 0.0009
	non-users (*n* = 183)	8.74%	91.26%	

**Table 4:** The use of dietary supplements OTHER than for stress, anxiety, depression, or sleeping problems among Wroclaw Medical University students in the academic year 2020/2021. Analyses were performed using the chi-square test.

The Use of Dietary Supplements OTHER than for Stress, Anxiety, Depression, or Sleeping Problems				
		YES	NO	*p*-Value
**Division**	Polish (*n* = 434)	68.43%	31.57%	*p* = 0.0000
	English (*n* = 190)	37.89%	62.11%	
**Gender**	Women (*n* = 463)	62.85%	37.15%	*p* = 0.0023
	Men (*n* = 157)	49.04%	50.96%	
**Faculty**	Medical (*n* = 320)	49.69%	50.31%	*p* = 0.0000
	Pharmacy (*n* = 189)	75.66%	24.34%	
	Dentistry (*n* = 53)	54.72%	45.28%	
	Health Science (*n* = 62)	61.29%	38.71%	
**Year of studies**	2nd (*n* = 166)	54.22%	48.75%	*p* = 0.0527
	3rd (*n* = 215)	57.21%	42.79%	
	4th (*n* = 116)	57.76%	42.24%	
	5th (*n* = 61)	65.57%	34.43%	
	6th (*n* = 66)	74.24%	25.76%	
**Level of studies**	pre-clinical (*n* = 497)	56.34%	43.66%	*p* = 0.0049
	clinical (*n* = 127)	70.08%	29.92%	
**Diet**	healthy diet (*n* = 341)	64.22%	35.78%	*p* = 0.0045
	unhealthy diet/difficult to say (*n* = 283)	53%	47%	
**The self-estimated stress level** **(0–10 pts scale)**	high, >6 pts (*n* = 449)	60.36%	39.64%	*p* = 0.1744
	moderate, 4–6 pts (*n* = 139)	58.99%	41.01%	
	low, <4 pts (*n* = 36)	44.44%	55.56%	
**The self-estimated knowledge about DS** **(0–10 pts scale)**	high, >6 pts (*n* = 247)	68.02%	31.98%	*p* = 0.0000
	moderate, 4–6 pts (*n* = 274)	62.04%	37.96%	
	low, <4 pts (*n* = 103)	30.1%	69.9%	
**DS for stress/anxiety/depression/sleeplesness**	users (*n* = 207)	67.63%	32.37%	*p* = 0.0024
	non-users (*n* = 417)	54.92%	45.08%	

**Table 5:** Correlation between different factors and the use of DS and POM. Analyses were performed using the logistic regression (OD—odds ratio, CI—confidence interval). * From other factors influencing the use of POM, checked in chi-square test but not included in the Table 5, significant impact of the use of DS for stress, anxiety, depression, or sleeping problems (*p* = 0.0001, OD: 0.27, CI: −0.16–0.52) was noticed.

Dependent Factors Independent Factors	The Use of Any DS	The Use of DS for Stress, etc.	The Use of DS Other than for Stress, etc.	The Use of POM for Stress, etc. *
	OR (95% CI)	OR (95% CI)	OR (95% CI)	OR (95% CI)
	*B* Coefficient	*B* Coefficient	*B* Coefficient	*B* Coefficient
	*p*-Value	*p*-Value	*p*-Value	*p*-Value
**Division** (ref Polish)	0.35 (0.21–0.59)	0.79 (0.48–1.30)	0.31 (0.19–0.50)	0.33 (0.16–0.66)
	−1.04	−0.24	−1.18	−1.12
	*p* = 0.0001	*p* = 0.3553	*p* = 0.0000	*p* = 0.0019
**Gender** (ref women)	0.71 (0.46–1.09)	0.70 (0.45–1.09)	0.65 (0.43–0.98)	0.40 (0.20–0.80)
	−0.34	−0.36	−0.43	−0.92
	*p* = 0.1206	*p* = 0.1117	*p* = 0.0380	*p* = 0.0091
**Faculty** (ref Medical)				
Pharmacy	1.19 (0.66–2.16)	0.69 (0.41–1.15)	1.46 (0.86–2.48)	0.57 (0.30–1.06)
	0.18	−0.37	0.38	−0.56
	*p* = 0.6544	*p* = 0.1570	*p* = 0.1569	*p* = 0.0756
Dentistry	1.17 (0.56–2.44)	1.01 (0.50–2.04)	0.95 (0.49–1.85)	0.66 (0.24–1.78)
	0.16	0.01	−0.05	−0.42
	*p* = 0.6673	*p* = 0.9741	*p* = 0.8882	*p* = 0.4095
Health Science	1.13 (0.52–2.45)	0.96 (0.46–1.97)	1.01 (0.52–2.06)	0.37 (0.12–1.08)
	0.13	−0.04	0.03	−0.99
	*p* = 0.7477	*p* = 0.9051	*p* = 0.9274	*p* = 0.0678
**Year of the Studies** **(ref 2nd Year)**				
3rd	1.30 (0.76–2.21)	1.25 (0.74–2.11)	1.30 (0.79–2.13)	1.92 (0.94–3.94)
	0.26	0.22	0.26	0.65
	*p* = 0.3372	*p* = 0.3909	*p* = 0.3043	*p* = 0.0751
4th	1.45 (0.79–2.66)	1.04 (0.58–1.87)	1.61 (0.91–2.84)	1.60 (0.69–3.71)
	0.37	0.04	0.47	0.47
	*p* = 0.2289	*p* = 0.8930	*p* = 0.1039	*p* = 0.2776
5th	2.38 (1.08–5.28)	1.89 (0.96–3.71)	2.10 (1.04–4.23)	1.71 (0.65–4.52)
	0.87	0.64	0.74	0.54
	*p* = 0.0325	*p* = 0.0654	*p* = 0.0373	*p* = 0.2761
6th	2.18 (0.98–4.86)	0.99 (0.48–2.01)	2.63 (1.27–5.46)	2.75 (1.09–6.95)
	0.78	−0.01	0.97	1.01
	*p* = 0.0546	*p* = 0.9696	*p* = 0.0095	*p* = 0.0328
**Unhealthy diet/difficult to say** **(ref healthy diet)**	0.74 (0.50–1.10)	1.52 (1.04–2.20)	0.68 (0.47–0.97)	1.63 (1.00–2.66)
	−0.30	0.42	−0.39	0.49
	*p* = 0.1357	*p* = 0.0288	*p* = 0.0332	*p* = 0.0494
**The self-estimated stress level**	1.13 (1.03–1.25)	1.24 (1.11–1.38)	(0.95–1.14)	1.26 (1.09–1.45)
	0.12	0.21	0.04	0.23
	*p* = 0.0141	*p* = 0001	*p* = 0.4101	*p* = 0.0021
**The self-estimated knowledge about DS**	1.31 (1.19–1.46)	1.35 (1.22–1.50)	1.15 (1.04–1.26)	1.11 (0.96–1.27)
	0.27	0.30	0.14	0.10
	*p* = 0.0000	*p* = 0.0000	*p* = 0.0037	*p* = 0.1487

## Data Availability

Data are available on request.

## Supplementary Materials

The following supporting information can be downloaded at: https://www.mdpi.com/article/10.3390/ijerph19127485/s1, Attachment S1: English-language version of the questionnaire. Table S1: Mean values of the year of the studies, stress, and knowledge levels in subgroups of students.
