# Supplementary material for: Factors That Influence the Use of Dietary Supplements among the Students of Wroclaw Medical University in Poland during the COVID-19 Pandemic
Source: Int J Environ Res Public Health. 2022 Jun 18;19(12):7485. doi: 10.3390/ijerph19127485 (PMC9224475; doi:10.3390/ijerph19127485)
Supplement: Supplementary file 1 [file ijerph-19-07485-s001.zip › Supplementary Attachment S1.pdf]

* Required

1. Q1. Gender *

Mark only one oval.

- female
- male
- other/prefer not to disclose

2. Q2. Major of studies (if you are a Student of more than one of the given majors of study, select the one in which you are studying at a higher year) *

Mark only one oval.

- medical
- dentistry
- pharmacy
- medical analytics
- dietetics
- physiotherapy
- nursing
- midwifery
- public health
- paramedic

3. Q3. Year of your studies *

Mark only one oval.

- 2nd
- 3rd
- 4th
- 5th
- 6th

4. Q4. Do you think your diet during the last academic year (2020/2021) was healthy (diverse and sufficient)? *

Mark only one oval.

- definitely yes
- rather yes
- I don't know /I cannot say
- rather not
- definitely not

5. Q5. How do you rate your personal stress level during the last academic year (2020/2021)? *

Mark only one oval.

	0	1	2	3	4	5	6	7	8	9	10	
lack of stress												extremely high stress

6. Q6. Did you take any dietary supplements and/or drugs available without prescription (over-the-counter drugs, OTC drugs) for stress/anxiety/depression/sleeping problems during the last academic year? *

Mark only one oval.

- yes *Skip to question 8*
- no *Skip to question 7*

Section 2

7. Q7. Why did not you take the dietary supplements and/or OTC drugs for stress/anxiety/depression/sleeping problems? More than one answer may be selected. *

Check all that apply.

- [ ] I did not need any dietary supplements or OTC drugs for stress/anxiety/depression/sleeping problems
- [ ] I do not believe that they are effective and can really help
- [ ] I do not believe that they are safe and do not cause any harm
- [ ] I only take drugs prescribed by the doctor
- [ ] I do not have enough knowledge about dietary supplements or OTC drugs

Other: [ ] ____________________

Skip to question 16

Section 3

8. Q8. What kind of dietary supplements and/or OTC drugs for stress/anxiety/depression/sleeping problems did you use during the last academic year? More than one answer may be selected. *

Check all that apply.

- [ ] ashwagandha (Withania somnifera)
- [ ] CBD oil (Cannabis sativa oil)
- [ ] ginseng (Panax ginseng)
- [ ] golden root (Rhodiola rosea)
- [ ] Gotu Kola (Centella asiatica)
- [ ] great basil (Ocimum basilicum)
- [ ] guarana (Paullinia cupana)
- [ ] honokiol (magnolia bark, Magnolia officinalis)
- [ ] hop (Humulus lupulus)
- [ ] kava kava (Piper methysticum)
- [ ] lion's mane mushroom (Hericium erinaceus)
- [ ] maca root (Lepidium peruvianum)
- [ ] magnolia-vine, schizandra (Schisandra chinensis)
- [ ] maypop, passionflower (Passiflora incarnata)
- [ ] melissa, lemon balm (Melissa officinalis)
- [ ] saffron (autumn) crocus (Crocus sativus)
- [ ] St John's-wort (Hypericum perforatum)
- [ ] valerian (Valeriana officinalis)
- [ ] water hyssop (Bacopa monnieri)
- [ ] wild chamomile (Matricaria recutita)
- [ ] B-group vitamins complex
- [ ] GABA
- [ ] glycine
- [ ] L-theanine
- [ ] magnesium/magnesium + vitamin B6
- [ ] melatonin
- [ ] tryptophan

Other: [ ] ______________________

9. Q9. What was the reason for using of above-mentioned dietary supplements and/or OTC drugs? More than one answer may be selected. *

Check all that apply.

- [ ] stress
- [ ] anxiety
- [ ] depression
- [ ] sleeping problems

10. Q10. How often did you use dietary supplements and/or OTC drugs for stress/anxiety/depression/sleeping problems during the last academic year? *

Mark only one oval.

- every day for the whole last academic year
- every day, but for a short time
- regularly before tests/exams
- a few times a week
- a few times a month
- sporadically, a few times a year
- Other: ____________

11. Q11. Did the pandemic situation with COVID-19 change the pattern of dietary supplements and/or OTC drug use when compared with the pre-pandemic time? More than one answer may be selected. *

Check all that apply.

- [ ] before the pandemic I did not take any, last year I used them
- [ ] I took them more often
- [ ] I took more different kinds of them
- [ ] I took greater doses of them
- [ ] I took them less often
- [ ] I took fewer different kinds of them
- [ ] I took lower doses of them
- [ ] nothing changed in their use

Other: [ ] ____________

12. Q12. Who has recommended you the dietary supplements and/or OTC drugs for stress/anxiety/depression/sleeping problems? More than one answer may be selected. *

Check all that apply.

- [ ] my own judgment
- [ ] general practitioner
- [ ] psychologist
- [ ] psychiatrist
- [ ] pharmacist
- [ ] nurse
- [ ] dietitian
- [ ] family member/friend/colleague
- [ ] Internet/social media
- [ ] television/radio
- [ ] medical books/professional journals
- [ ] non-medical materials (e.g., books, newspapers, leaflets)

Other: [ ] ____________

13. Q13. Where did you buy dietary supplements and/or OTC drugs for stress/anxiety/depression/sleeping problems? More than one answer may be selected. *

Check all that apply.

- [ ] pharmacy/drugstore
- [ ] pharmacy/drugstore on the Internet
- [ ] supermarkets, small shops, gas station etc.
- [ ] shops with healthy food and/or dietary supplements
- [ ] online shops with healthy food and/or dietary supplements
- [ ] directly from manufacturer (stationary, sales representative or online)

Other: [ ] ______________________

14. Q14. How do you estimate the efficacy of taken dietary supplements and/or OTC drugs for stress/anxiety/depression/sleeping problems? *

Mark only one oval.

	0	1	2	3	4	5	6	7	8	9	10	
completely ineffective	( )	( )	( )	( )	( )	( )	( )	( )	( )	( )	( )	fully effective

15. Q15. Have you ever experienced any adverse effects that you would combine with the use of dietary supplements and/or OTC drug for stress/anxiety/depression/sleeping problems? More than one answer may be selected. *

Check all that apply.

- [ ] I have never experienced any adverse effects
- [ ] nausea, vomiting, loss of appetite
- [ ] stomachache, heartburn
- [ ] diarrhea, bloating
- [ ] constipation
- [ ] headache and/or dizziness
- [ ] fatigue, sleepiness
- [ ] problems with concentration
- [ ] anxiety, agitation, sleeplessness
- [ ] problems with breathing, asthma attacks
- [ ] itching, rash, urticaria
- [ ] heart palpitations/irregular heart rate
- [ ] too low blood pressure
- [ ] too high blood pressure
- [ ] sexual dysfunction
- [ ] worsening of laboratory check results

Other: [ ] ______________________

Section 4

16. Q16. Did you take any drugs prescribed by a general practitioner and/or specialist (e.g., psychiatrist) for stress/anxiety/depression/sleeping problems during the last academic year? *

Mark only one oval.

- yes, regularly the whole academic year
- yes, sometimes e.g., before tests/exams
- no, never
- prefer not to disclose

17. Q17. Did you take any dietary supplements and/or OTC drugs OTHER than for stress/anxiety/depression/sleeping problems during the last academic year? *

Mark only one oval.

- yes
- no *Skip to question 20*

Section 5

18. Q18. What was the reason to take dietary supplements and/or OTC drugs OTHER than for stress/anxiety/depression/sleeping problems during the last academic year? More than one answer may be selected. *

Check all that apply.

- for beauty hair, skin, nails
- to improve training regimens
- to improve immunity/stay healthy
- to improve sexual performance
- to supplement vegan/vegetarian diet
- to increase cognition, concentration, and alertness
- to increase energy and physical performance
- to increase muscle mass
- to lose weight and/or decrease fat mass
- to prevent or treat chronic or acute diseases
- to prevent or treat microelements/nutrients deficiency
- to protect joints
- to protect/to improve liver function
- to supplement a medical elimination diet

Other: ________________

19. Q19. What kind of dietary supplements and/or OTC drugs OTHER than for stress/anxiety/depression/sleeping problems have you taken during the last academic year? More than one answer may be selected. *

Check all that apply.

- [ ] beta-alanine
- [ ] calcium
- [ ] collagen
- [ ] creatine
- [ ] folic acid
- [ ] glucosamine
- [ ] glutamine
- [ ] guarana (Paullinia cupana)
- [ ] iron
- [ ] multivitamins
- [ ] omega-3 fatty acids
- [ ] pre- or probiotics
- [ ] protein powder, shakes or other products
- [ ] spirulina
- [ ] vitamin B12
- [ ] vitamin C
- [ ] vitamin D/vitamin D + MK7
- [ ] vitamin E/vitamin A+E
- [ ] vitaminized water/beverages
- [ ] yerba mate
- [ ] white mulberry (Morus alba)
- [ ] zinc

Other: [ ] ____________________

Section 6

20. Q20. How do you rate your knowledge about dietary supplements? *

Mark only one oval.

	0	1	2	3	4	5	6	7	8	9	10	
I do not know anything	○	○	○	○	○	○	○	○	○	○	○	I think I know very much

21. Q21. Have you learned about dietary supplements during your studies? *

Mark only one oval.

- ○ yes, I have learned a lot
- ○ yes, I have learned enough
- ○ yes, I have learned a little bit
- ○ not yet, but I probably will
- ○ not yet, and I do not know if I will
- ○ I do not remember such topics

22. Q22. If you are now a final-year student, do you think that you should have learned more about dietary supplements during your studies? *

Mark only one oval.

- ( ) I am not a final-year student yet
- ( ) yes, much more as an obligatory subject
- ( ) yes, much more in optional classes
- ( ) yes, a little bit more but as an obligatory subject
- ( ) yes, a little bit more but only in optional classes
- ( ) no, it was enough
- ( ) no, it is unnecessary
- ( ) I have no opinion
- ( ) Other: ____________________

This content is neither created nor endorsed by Google.

Google Forms
