# Supplementary material for: Factors That Influence the Use of Dietary Supplements among the Students of Wroclaw Medical University in Poland during the COVID-19 Pandemic
Source: Int J Environ Res Public Health. 2022 Jun 18;19(12):7485. doi: 10.3390/ijerph19127485 (PMC9224475; doi:10.3390/ijerph19127485)
Supplement: Supplementary file 1 [file ijerph-19-07485-s001.zip › Supplementary Table S1.pdf]

Table S1: Mean values of the year of the studies, stress, and knowledge levels in subgroups of students.
Analyses were performed using U-Mann Whitney or Kruskal-Wallis tests (for two or more groups, respectively) with Dunn's *post-hoc* test and Bonferroni correction.

		Year of studies mean ± SD	p-value	Stress level mean ± SD	p-value	Level of knowledge mean ± SD	p-value
**Division**	Polish	3.30 ± 1.26	**p = 0.0000**	7.21 ± 2.07	p = 0.6308	6.00 ± 2.02	**p = 0.0002**
	English	3.73 ± 1.24		7.35 ± 1.87		5.33 ± 2.16	
**Gender**	Women	3.38 ± 1.29	**p = 0.0279**	7.43 ± 1.89	**p = 0.0007**	5.83 ± 2.06	p = 0.9616
	Men	3.60 ± 1.21		6.70 ± 2.27		5.78 ± 2.12	
**Faculty**	Medical (M)	3.53 ± 1.21	**p = 0.0000** HS *vs.* M HS *vs.* Ph HS *vs.* D	6.99 ± 2.04	**p = 0.006** Ph *vs.* M	5.44 ± 2.11	**p = 0.0000** Ph *vs.* M **p = 0.0127** Ph *vs.* HS
	Pharmacy (Ph)	3.48 ± 1.42		7.61 ± 1.82		6.47 ± 2.0	
	Dentistry (D)	3.72 ± 0.93		7.42 ± 2.27		5.83 ± 1.93	
	Health Science (HS)	2.53 ± 0.94		7.32 ± 1.93		5.55 ± 1.88	
**Year of studies**	2nd	-------------	-------------	7.52 ± 1.83	**p = 0.0426** 6th *vs.* 2nd	5.32 ± 2.0	**p = 0.0015** 5th *vs.* 2nd **p = 0.0095** 5th *vs.* 3rd **p = 0.0000** 6th *vs.* 2nd and 3rd **p = 0.0447** 6th *vs.* 4th
	3rd	-------------		7.07 ± 2.09		5.51 ± 2.20	
	4th	-------------		7.53 ± 1.68		5.97 ± 1.79	
	5th	-------------		7.36 ± 2.30		6.57 ± 1.88	
	6th	-------------		6.58 ± 2.27		6.88 ± 2.01	
**Year of studies**	pre-clinical	-------------	-------------	7.33 ± 1.93	p = 0.2567	5.56 ± 2.06	**p = 0.0000**
	clinical	-------------		6.95 ± 2.31		6.73 ± 1.95	
**Diet**	healthy diet	3.55 ± 1.30	**p = 0.0143**	6.91 ± 2.0	**p = 0.0000**	6.02 ± 2.04	**p = 0.0018**
	unhealthy diet/difficult to say	3.29 ± 1.22		7.66 ± 1.95		5.53 ± 2.12	
**The self-estimated stress level (0-10 pts scale)**	high, > 6 pts (H)	3.39 ± 1.25	p = NS in all comparisons	-------------	-------------	6.02 ± 2.03	**p = 0.0001** H *vs.* M
	moderate, 4-6 pts (M)	3.47 ± 1.28		-------------		5.13 ± 2.07	
	low, < 4 pts (L)	2.86 ± 1.40		-------------		5.50 ± 2.31	
**The self-estimated knowledge about DS (0-10 pts scale)**	high, > 6 pts (H)	3.75 ± 1.37	**p = 0.0002** H *vs.* L	7.56 ± 1.88	**p = 0.0000** H *vs.* L **p = 0.0028** H *vs.* M	-------------	-------------
	moderate, 4-6 pts (M)	3.28 ± 1.19		7.25 ± 1.98		-------------	
	low, < 4 pts (L)	3.75 ± 1.37		6.50 ± 2.21		-------------	
**DS for stress/anxiety/ depression/sleeplesness**	users	3.54 ± 1.28	p = 0.1428	7.89 ± 1.80	**p = 0.0000**	6.61 ± 1.92	**p = 0.0000**
	non-users	3.38 ± 1.26		6.93 ± 2.08		5.39 ± 2.05	
**Other DS**	users	3.55 ± 1.32	**p = 0.0137**	7.32 ± 1.87	p = 0.6691	6.18 ± 1.88	**p = 0.0000**
	non-users	3.26 ± 1.17		7.15 ± 2.21		5.23 ± 2.24	
**Any dietary supplement**	users	3.53 ± 1.30	**p = 0.01**	7.42 ± 1.87	**p = 0.0083**	6.24 ± 1.88	**p = 0.0000**
	non-users	3.21 ± 1.16		6.86 ± 2.27		4.77 ± 2.19	
**POM**	no, never (N)	3.41 ± 1.26	NS in all comparisons	7.03 ± 1.99	**p = 0.0002** YS *vs.* N **p = 0.0132** YR *vs.* N **p = 0.0002** PD *vs.* N	5.65 ± 2.10	**p = 0.0016** YR *vs.* N
	yes, sometimes (YS)	3.61 ± 1.68		7.82 ± 2.25		6.39 ± 1.53	
	yes, regularly (YR)	3.67 ± 1.33		8.24 ± 1.61		6.65 ± 2.12	
	prefer not to disclose (PD)	3.0 ± 1.15		8.84 ± 1.21		5.74 ± 2.10	
